# Transcutaneous electrical acupuncture stimulation (TEAS) for gastrointestinal dysfunction in adults undergoing abdominal surgery: study protocol for a prospective randomized controlled trial

**DOI:** 10.1186/s13063-020-04470-4

**Published:** 2020-07-06

**Authors:** Ya-Fan Bai, Chao Gao, Wen-Jing Li, Yi Du, Li-Xin An

**Affiliations:** 1 Department of Anesthesiology, Beijing Friendship Hospital, Capital Medical University, No. 95 Yongan Road, Xicheng District, Beijing, 100050 China; 2 Department of Anesthesiology, Beijing Huimin Hospital, Beijing, China; 3 Department of Traditional Chinese Medicine, Beijing Friendship Hospital, Capital Medical University, Beijing, China

**Keywords:** TEAS, Postoperative gastrointestinal function, Randomized controlled trials, Protocol

## Abstract

**Background:**

Postoperative gastrointestinal (GI) dysfunction (PGD) is a common problem after abdominal surgery. PGD can increase the length of hospital stay and may lead to serious complications. Acupuncture and moxibustion are alternative therapies for PGD that have been used in some settings. However, the effect of preventive application of acupuncture or transcutaneous electrical acupuncture stimulation (TEAS) is still uncertain. The purpose of this study is to investigate the efficacy of the continuous application of TEAS on GI function recovery in adults undergoing abdominal surgery. At the same time, we will try to confirm the mechanism of TEAS through the brain–gut axis.

**Methods/design:**

This study is a prospective, single-center, two-arm, randomized controlled trial that will be performed in a general hospital. In total, 280 patients undergoing abdominal surgery were stratified by type of surgery (i.e. gastric or colorectal procedure) and randomized into two treatment groups. The experimental group will receive TEAS stimulation at L14 and PC6, ST36 and ST37. The sham group will receive pseudo-TEAS at sham acupoints. The primary outcome will be the time to the first bowel motion by auscultation. The recovery time of flatus, defecation, the changes in perioperative brain–intestinal peptides, postoperative pain, perioperative complications, and hospitalization duration will be the secondary outcomes.

**Discussion:**

The results of this study will demonstrate that continuous preventive application of TEAS can improve the GI function recovery in patients undergoing abdominal surgery and that this effect may act through brain–gut peptides.

**Trial registration:**

Chinese Clinical Trial Registry, ChiCTR1900023263. Registered on 11 May 2019.

## Introduction

Postoperative gastrointestinal dysfunction (PGD) refers to a series of clinical syndromes in which digestive tract symptoms are the main manifestation after a surgical operation. The most common postoperative syndromes affect the gastric, intestinal, and biliary tract systems, and patients often experience symptoms such as abdominal distension, constipation, nausea and vomiting, and defecation disorders. The incidence of PGD can be as high as 10%–30% [1, 2]. Despite ongoing research and different new drug treatments, the incidence of abdominal distention within 24 h of an operation is 8%–28% of all surgeries [3]. Patients with PGD have a longer duration of hospitalization, heavier economic burdens, and a much higher risk of serious complications, such as infection, electrolyte disturbances, and even life-threatening conditions [3–5]. Therefore, for postoperative patients in the hospital, restoring the normal function of the gastrointestinal (GI) tract as soon as possible for postoperative rehabilitation is a very important step.

Once PGD appears, the main treatment measures that can be taken include the use of gastric dynamics promoting drug, GI decompression, nutritional support, non-steroidal anti-inflammatory drug treatments, and so on [2, 6]. However, the therapeutic effect of these treatments is limited, and the patients’ satisfaction rate is relatively low [6]. Currently, many studies have demonstrated that acupuncture and moxibustion have unique advantages in the treatment of PGD [7] and have gradually obtained more acceptance from physicians as an alternative therapy.

Recently, the brain–gut axis, serving as a bridge between the central nervous system (CNS) and the GI tract [8, 9], has been proved to be one of the possible mechanisms of acupuncture improving GI. Through the brain–gut axis, signals from the brain affect the sensory, motor, and endocrine modalities, as well as the microflora of the GI gut. Similarly, visceral information from the microbiota may also influence brain functions [8]. In our previous studies, our research group also found that electroacupuncture stimulation of Zusanli (ST36) could lead to imaging changes in different brain regions observed by functional magnetic resonance imaging (fMRI) [10]. These results demonstrated that acupuncture may act through the brain–gut axis. Brain–gut peptides are distributed along the GI tract and in the CNS and are involved in the modulation of GI tract processing [11]. The secretion of substance P (SP) and vasoactive intestinal polypeptide (VIP) are decreased, motilin and cholecystokinin (CCK) are increased after treatment with electrical acupuncture (EA) in rats with irritable bowel syndrome (IBS) [12, 13]. However, until now, most research on this mechanism has focused on animal studies and the treatment effect of acupuncture or transcutaneous electrical acupuncture stimulation (TEAS) on patients after abdominal surgery—whether the prevention effect of TEAS on PGD through brain–gut peptides or not—is still unclear.

Based on previous studies, our aim is to confirm the preventive effect of TEAS on PGD in patients undergoing abdominal surgery. Changes in the perioperative brain–gut peptides may prove that the regulation of GI function by TEAS stimulation plays a role through the brain–intestinal axis. Health economic indicators (length of stay, cost of hospitalization, etc.), will also demonstrate the significance of perioperative TEAS stimulation on postoperative GI function recovery of patients undergoing abdominal surgery. This study will provide further evidence as to whether TEAS should be included in enhanced recovery after surgery (ERAS) protocols for patients undergoing GI surgery.

## Methods

The protocol for this trial is reported based on the Standard Protocol Items: Recommendations for Interventional Trials (SPIRIT) 2013 Checklist: defining standard protocol items for clinical trials (Additional file 1). The study has been approved by the Ethics Committee of Beijing Friendship Hospital (approval number 2019-P-065-01) and has been registered in the Chinese Clinical Trial Registry (Chictr) (registration number ChiCTR1900023263). This study is still in process.

### Trial design

**Fig. 1:**
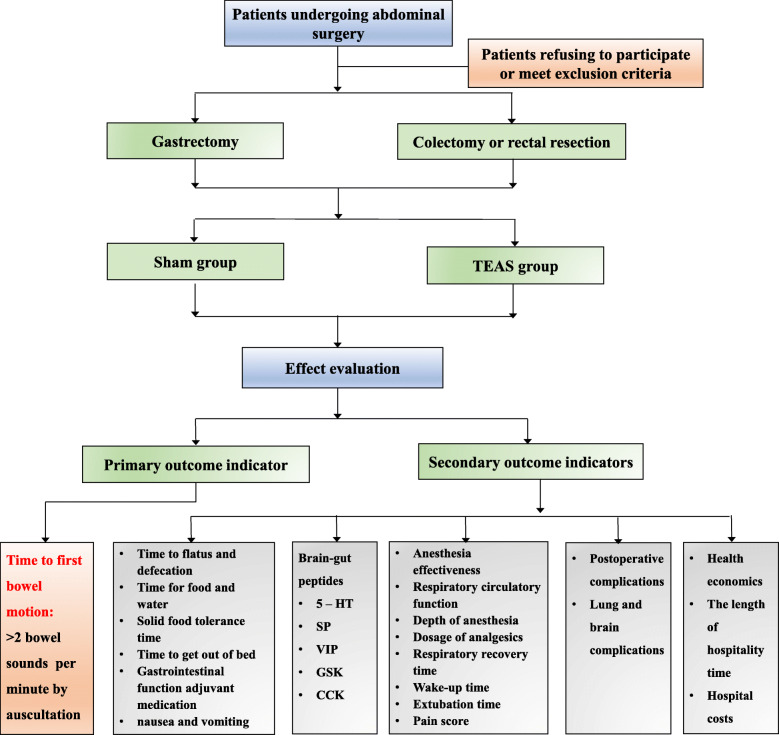
Consolidated Standards of Reporting Trials (CONSORT) diagram for this trial

The trial is a single-center, prospective, randomized controlled trial (RCT) with a two-arm, parallel-group design (Fig. 1). Data analysis will be performed according to the superiority principle. The study will be carried out at Beijing Friendship Hospital and will be performed according to the principles of the Declaration of Helsinki (version Edinburgh 2000).

The study will continue for 24 months, and all the selected individuals will be stratified by type of surgery (i.e. gastric or colorectal procedure) and randomized into two groups: the TEAS treatment group (Group T) and the sham group (Group S). The researchers will conduct screening in accordance with the established criteria and pre-standard treatment plan. Data collection will start from the collection of basic data until the end of follow-up (Table 1).

**Table 1:**
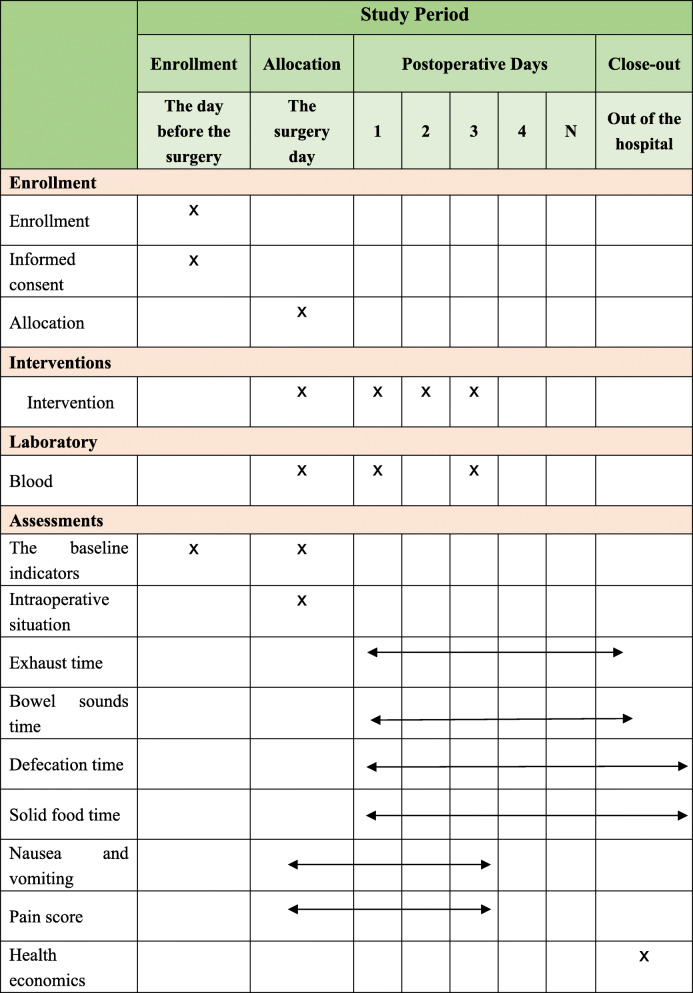
Standard Protocol Items: Recommendations for Interventional Trials (SPIRIT) Schedule for enrollment, interventions, and assessments

### Randomization and blinding

This is a single-blinded study. The doctors from the traditional Chinese department who will conduct the TEAS stimulation and the anesthesiologists will know the grouping information and participants. The observers of major outcomes after surgery will be blinded. Randomization will be conducted by a computer-generated blocked randomization list with 70 blocks of four patients per block. The participants will be consecutively randomly assigned to the TEAS group or the sham group at a ratio of 1:1. Allocation shall proceed via duplicate, sealed, numbered envelopes, which will be stored by the clinical trial quality control inspectors and the main person in charge of this research group. The blind bottom shall not be opened without reason during the trial. The outcome assessors and the statisticians will not participate in the treatment, they will perform the outcome evaluation and the statistical analysis independently.

### Study participants and recruitment

We will recruit 280 patients scheduled to undergo laparoscopic resection of gastric and colorectal tumors. These patients will be recruited from Beijing Friendship Hospital affiliated with Capital Medical University after they meet the eligibility criteria and sign their informed consent. We plan to enroll the first patient on July 1, 2019 and to end on December 31, 2020. All participants will sign the informed consent form for participating in the clinical trial and the informed consent form for collecting blood samples.

#### Inclusion criteria

Inclusion criteria are as follows: (1) aged 18–70 years, regardless of gender; (2) undergoing endotracheal intubation and laparoscopic abdominal tumor resection under general anesthesia (stomach, colon, and rectum); (3) body mass index (BMI) 18–30 kg/m^2^ (BMI = weight in kilograms / height in meters squared); (4) American Society of Anesthesiologists (ASA) classification levels I–III; (5) requirement for a postoperative analgesia pump and signing of informed consent; and (6) volunteering to participate in this study and sign the informed consent.

#### Exclusion criteria

Exclusion criteria are as follows: (1) surgical incision or scar on the meridian of L14 / PC6 / ST36 / ST37; (2) local skin infection at acupoints; (3) upper limb or lower limb nerve injury; (4) a history of spinal surgery; (5) participation in other clinical trials within the past 4 weeks; (6) inability to understand the visual analog scale (VAS) score and numeric rating scale (NRS) score or to use patient-controlled analgesia (PCA); (7) presence of a pacemaker; (8) preoperative complications involving severe CNS diseases or severe mental diseases; (9) operations requiring enterostomy or conversion to open surgery; or (10) necessity of transfer to the intensive care unit (ICU) for treatment after surgery.

#### Discharge criteria

Discharge criteria are as follows: (1) requirement for individuals to withdraw during the test; (2) violation of the test program; and (3) occurrence of serious adverse events (AEs).

### Interventions

**Fig. 2:**
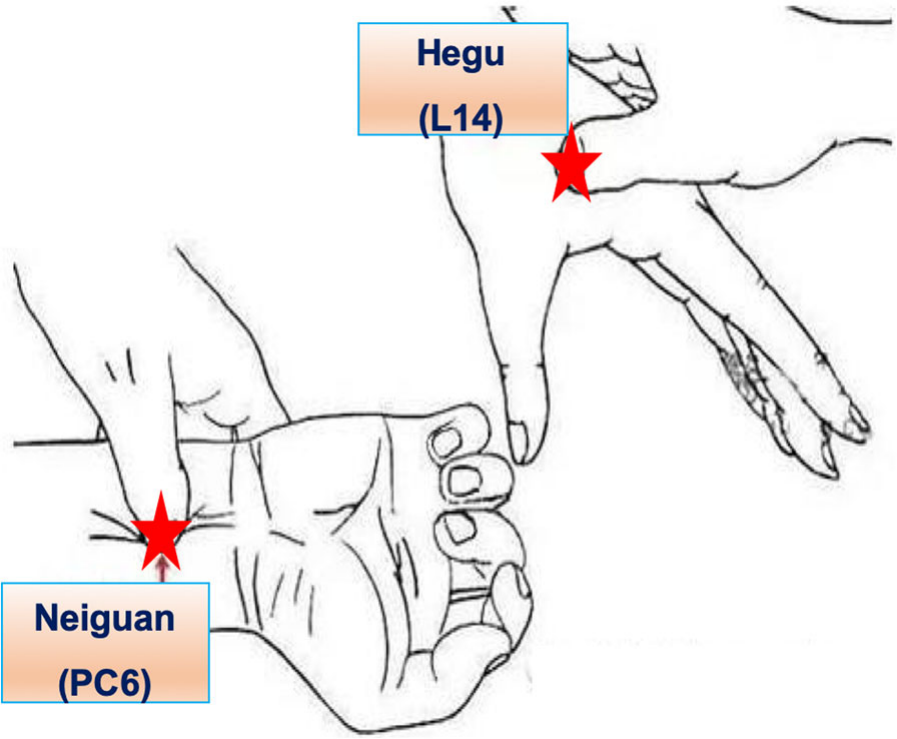
The location of L14, PC6. L14 is the Hegu acupoint; PC6 is the Neiguan acupoint

**Fig. 3:**
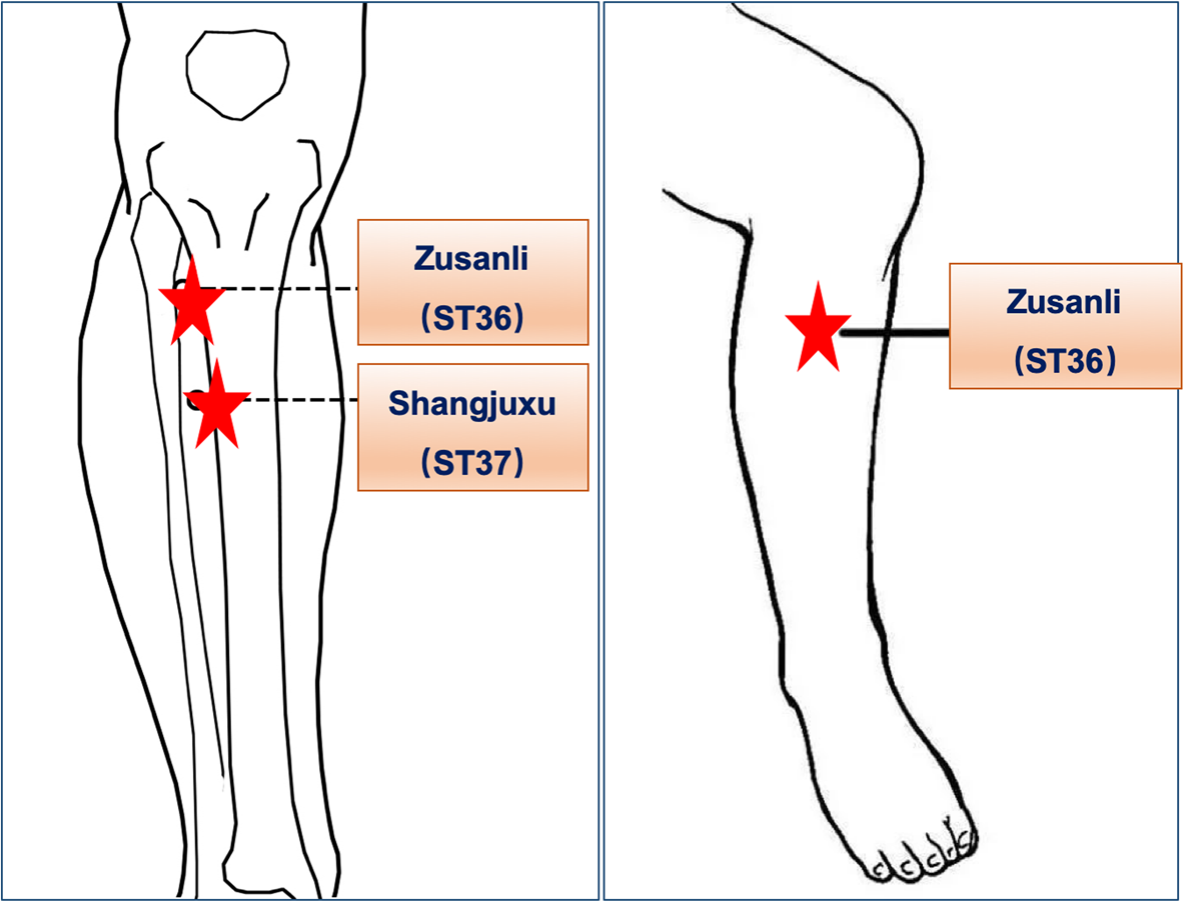
The location of ST36, ST37. ST36 is the Zusanli acupoint; ST37 is the Shangjvxu acupoint

Patients receiving TEAS treatment will be treated bilaterally at four acupoints: Hegu (L14) and Neiguan (PC6), as well as Zusanli (ST36) and Shangjuxu (ST37). L14 is an acupoint of the hand yangming large intestine meridian. It is located on the dorsum of the hand between the first and second metacarpal bones (Fig. 2). PC6 belongs to the pericardium meridian. It is located between the flexor carpi radialis muscle tendon and the palmaris longus tendon, 2 cun above the rasceta (Fig. 2). ST36 and ST37 are acupoints of the foot yangming stomach meridian. ST36 is located on the lateral side of the lower leg, 3 cun below Dubi and one finger width lateral to the anterior border of the tibia (Fig. 3). ST37 is located 3 cuns below ST36. An electrode with wire will be attached to the site of these acupoints. The wire is connected to the HANS acupoint nerve stimulator (HANS-200A, Nanjing Jisheng Medical Technology Co., Ltd., China). TEAS stimulation uses a dense-dispersed wave with frequencies of 2 and 100 Hz alternating every 3 s. The waveform is a symmetric biphasic curve. The stimulation intensity will be set to the level of maximum tolerance for each patient. During the operation, the TEAS stimulation will span from before the induction to the end of the operation. On the first, second, and third days after surgery, the acupuncture doctor will arrive at the ward in the morning at 09:00 to administer TEAS stimulation at the same acupoints for 30 min. This stimulation will be continued until the patients’ flatus recovery.

**Fig. 4:**
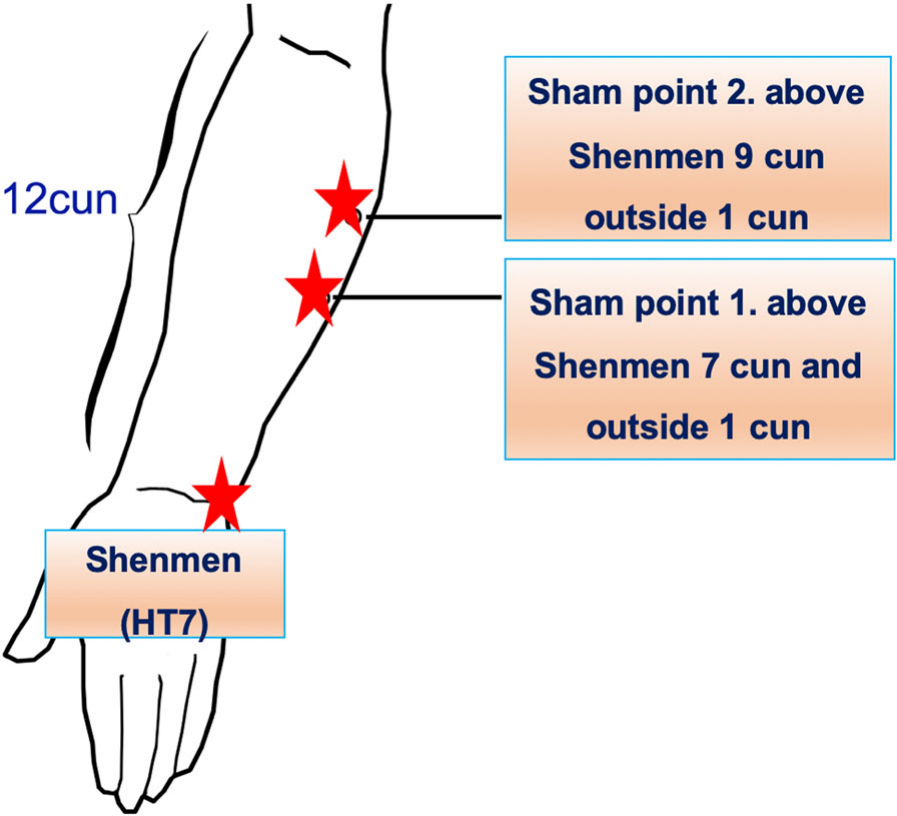
The location of sham points 1 and 2. HT7 is the Shenmen acupoint. Cun is the unit of length

**Fig. 5:**
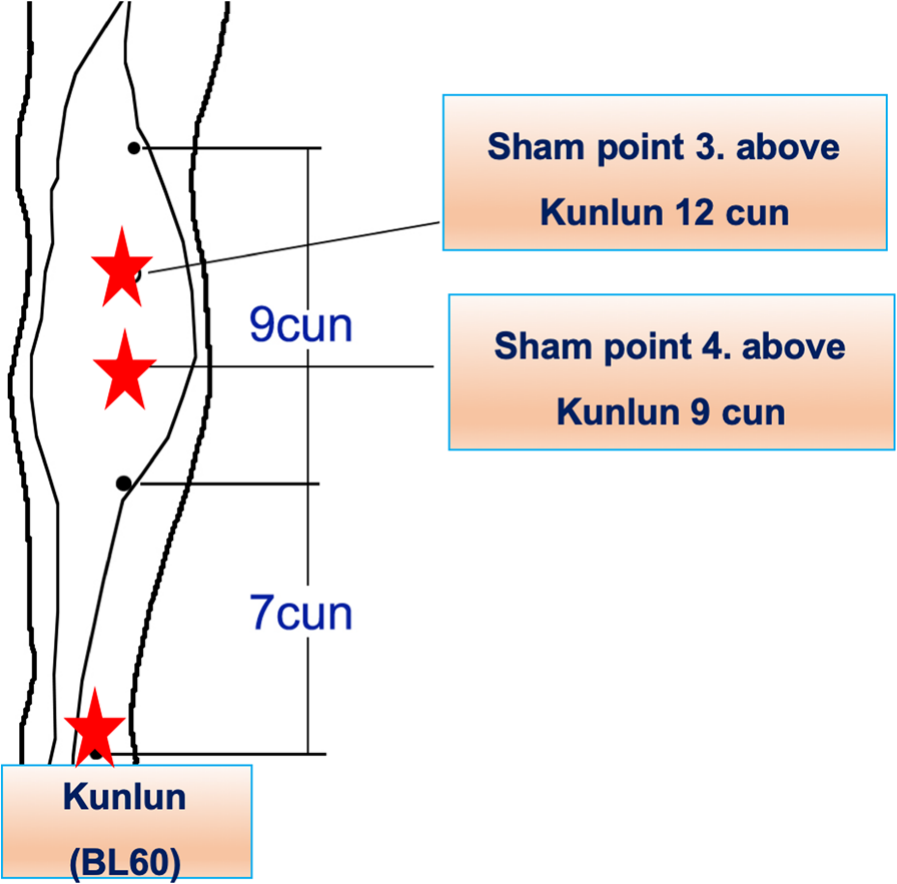
The location of sham points 3 and 4. BL60 is the Kunlun acupoint. Cun is the unit of length

In the sham group, the sham points are at 7 cun and 9 cun above Shenmen (HT7) and outside 1 cun (Fig. 4). The other two sham points are at 9 cun and 12 cun above the Kunlun (BL60) (Fig. 5). There are no meridian and channels through these four sites; thus, we will use these positions as our sham points. We will put the electrode on the sham points and connect it to the HANS stimulator but not administer any stimulation. The same doctor will go to the ward, put the electrode on these points, and connect it to the stimulator (not open) at the same time after surgery, until the first flatus recovery.

The anesthesia is total intravenous anesthesia; no epidural, paravertebral block, TAP, or other nerve block is added during the operation. Before the end of the operation, 0.5% ropivacaine incision infiltration block will be performed in the incision of endoscope. The general anesthesia drugs and methods during the operation are determined by the chief anesthesiologist and conform to the treatment routine and work habits.

### Postoperative analgesia measures and remedial plan

The analgesic pump configuration: after the operation, the intravenous analgesic pump was connected and the analgesic formula sufentanil 250 μg was added with normal saline to 250 mL, 2 mL/h, and PCA 3 mL at an interval of 15 min. (1) Analgesia recovery: participants were given Flubiprofen 50 mg/intravenous glucose tolerance test in the ward when the pain VAS score was ≥ 4 points and the pain score was still ≥ 4 points after five consecutive effective PCA compressions. (2) Remedy of nausea and vomiting: the NRS method was used for nausea score. When the nausea score was ≥ 7 points or vomiting ≥ 1 time, serotonin 3 receptor antagonist was given. The remedial medication should be recorded in the remedial medication list. (3) Concomitant medication record: concomitant medication during the trial period (from the start of surgery to 5 days after the end of surgery).

### Outcome assessment

A blind observer from the Data Monitoring Committee (DMC) will collect all outcome data after surgery. The primary outcome of this study will be time to the first bowel motion by auscultation. The same surgical residents will listen to the bowel sounds every 4 h postoperatively. Time to first bowel motion is the duration until regular bowel sounds (more than two sounds every minute) are first heard after surgery [14].

The secondary endpoints will include the time to first flatus and time to defecation. This information will be obtained from participant questionnaires filled out once daily with the help of nurse staff. To observe changes in plasma brain–gut peptides, such as 5-HT, SP, VIP, gastrin, and CCK, we will collect venous blood from patients before the operation and on the first and third days after the operation. We will then centrifuge and freeze the plasma supernatant for later testing. We cooperate with our clinical experimental center to collect, code, centrifuge, freeze store, store, and test blood samples by a professional team. Secondary endpoints will also include VAS score of postoperative pain, vomiting and nausea, length of stay in the ICU, length and cost of stay in the hospital, as well as clinical complications such as fever, pneumonia, wound infection, and bleeding. Secondary outcome will be assessed by the medical team. The length of hospital stay is calculated as the number of days from the date of the operation to the date of discharge. Criteria for hospital discharge will include stable vital signs, the ability to tolerate solid foods after defecation without nausea or vomiting, and the occurrence of no other complications after the operation.

### Sample size calculation

Due to a lack of related reports on the preventive usage of TEAS in patients undergoing GI surgery, we evaluated the effectiveness of TEAS according to our pre-experimental results. A priority-effectiveness test was used and the ratio between the groups was 1:1. According to the pre-experimental results, a difference in time to first bowel sounds of 12 h was thought to be clinically significant and selected as the effect size. This result in a hazard ration of 1.5. The COX regression sample size calculation formula was used to complete the sample size estimation. We set unilateral α = 0.05 and power = 0.90; PASS 2011 software was used for sample estimation and a total of 256 effective cases were estimated. According to the above sample estimation result and assuming 10% patient loss, a minimum of 280 cases is required. Therefore, we have set the required number of cases in this study to 280 cases, including 140 cases in the TEAS group and 140 cases in the sham TEAS group. Our General Surgery Department performs 400–500 cases of GI surgery per year; consequently, this study period is set at 2 years.

### Statistical methods

Statisticians are responsible for negotiating with major researchers to develop statistical analysis plans, establish databases, and conduct statistics with the SPSS statistical analysis system. All data will be collated by statisticians. The intention processing principle in SPSS 22.0 will be used for the data analysis. A unilateral test will be used for statistical results, and a *P* value < 0.05 will be considered statistically significant. The results will be expressed as the mean ± standard deviation. The t-test will be used for the normality and homogeneity of data, the hypothesis test of superiority will be used for major outcome indicators, the chi-square test will be used for normal distribution data, and the rank sum test or Fisher’s exact probability method will be used for non-normal distribution data. Statistical methods for the main efficacy indicators have been determined by the Capital Medical University statistical department.

### Data collection methods and monitoring

The statistical professionals are responsible for formulating the statistical analysis plan through consultation with the main researchers, establishing the database, and using the SPSS statistical analysis system for statistics. A comprehensive efficacy analysis was conducted in accordance with the program set, and the whole analysis set, demographic and other baseline characteristics, and other efficacy indicators were analyzed in accordance with the program set.

The DMC is consisting by a doctor in charge of data collection and sorting, a scientific research manager and a statistician. The doctor from the DMC will record the actual number of individuals enrolled, the cases of exclusion, demographic and other baseline characteristics, compliance analysis, safety analysis, incidence of complications and combined treatment, and comprehensive efficacy evaluation. The demographic characteristics, medical history, and treatment history of the patients will be described, including the comparison of age, gender, disease course, and disease condition at the time of enrollment. The scientific research management committee will have access to the final trial dataset. At the end of the study, the original data and results will be submitted to the scientific research management committee; they will be disclosed to the public after the results are published.

### Safety evaluation

AEs are the appearance or progression of any symptoms of discomfort, syndromes, or diseases that occur during a clinical trial and affect the health of the individual. Any abnormalities in clinical testing that indicate the presence of disease and/or organ toxicity and serious abnormalities requiring active treatment (such as withdrawal of medication, increased follow-up, and diagnostic studies) are considered AEs. In the process of clinical research, researchers should fill in the AE record form truthfully and in detail, recording the clinical manifestations, occurrence time, severity, duration, measures taken, and outcomes of AEs.

When an AE occurs, the observing physician may decide whether to suspend the observation or not according to the condition. All AEs should be tracked and documented in detail until the individual’s situation is properly resolved or the individual is in a stable condition; if laboratory tests are abnormal and clinically significant, they should be tracked back to pretreatment levels.

## Discussion

PGD comprises the most common series of clinical syndromes with digestive symptoms as the main manifestation after surgery and is commonly seen after abdominal surgery; the delay of flatus and defecation is often the first sign of PGD in patients [1, 2]. The application of traditional Western medicine has corresponding defects and the results are often unsatisfactory [6]. According to the theory of traditional Chinese medicine, PGD is one of the diseases of “intestinal bi” and “intestinal knot.” Abdominal surgical injury and meridian blood vessels, blood stasis to block intestinal fu, intestinal fu internal turbid qi stasis, and tangible evil obstruction result in fu-qi obstruction. After the operation, qi and blood deficiency, adverse transport, loss of GI function, plus surgery and injury to flesh, muscles, and bones result in bleeding outside the vein, block qi, and cause abdominal pain, abdominal distension, nausea, vomiting, and a series of symptoms.

A large number of previous clinical studies and animal experiments have confirmed that the effect of electroacupuncture and TEAS can improve postoperative GI disorders [15, 16]. Electrical stimulation of Zusanli can activate vagus nerve fibers by a spinal cord reflex and promote gastric motility [17]. Many clinical studies have found that EA could stimulate patients’ gastrointestinal motility, which is why we set the time to first bowel motion as the primary outcome of this study [14, 17]. Similarly, it has been reported by Iwa et al. [18] that the application of EA in rats with conscious free movement may promote distal colon movement and accelerate colon transport through the sacral efferent parasympathetic pathway. EA may help repair damage to the GI barrier by regulating the neuroendocrine immune system and fighting inflammation [19]. EA stimulation of Zusanli and other acupoints can play a protective role in intestinal injury and mucosal barrier dysfunction of rats with blood loss by activating cholinergic anti-inflammatory pathways and intestinal glial cells (known to secrete epidermal growth factor, thereby increasing the recovery rate of epithelial cells) [20–22].

In recent years, research on the brain–gut axis has become a hot topic. The brain affects the sensation, movement, endocrine, and microflora of the GI tract through the brain–gut axis. Gut information from the microbiome may also affect brain function [8, 9, 23]. Brain–gut peptide, regulation of the vagus nerve, and nerve excitability of the posterior horn of the spinal cord are all possible mechanisms of the brain–gut axis regulating GI function [24]. Brain–gut interaction is achieved by a variety of substances in the endocrine cells of the CNS, neuroendocrine system, and GI tract, which act as neurotransmitters and hormones at the same time and are called brain–gut peptides [25]. Brain–gut peptides widely regulate GI activity and are therefore closely associated with PGD. Major excitatory neurotransmitters include histamine, 5-HT, SP, calcitonin gene-related peptide (CGRP), and adrenocorticotropin releasing factor-related peptide (CRF). Major inhibitory neurotransmitters include cholecystokinin (CCK), nitric oxide (NO), norepinephrine (NE), and VIP [26]. Clinical studies have confirmed that the contents of 5-HT in the intestinal mucosa of patients with GI dysfunction are significantly increased. Other studies have found that EA can regulate the secretion of SP, SP receptor, and VIP in the IBS model colon [27]. CRF can induce repeated defecation in rats. Both sets of findings have confirmed the possible role of brain–gut peptides in patients with GI dysfunction [28].

Although the clinical therapeutic effect of electroacupuncture on GI dysfunction has been confirmed by many investigations, the role of preventive acupuncture treatment of perioperative patients, especially high-risk patients (receiving abdominal surgery), and the mechanism of this role associated with the brain–intestinal tract have not been confirmed by any clinical studies and still need to be verified by much research.

However, some limitations in this study should be noted. First, the main outcome measures are subjective evaluation scales that lack objective evidence to directly support the effect of a given treatment. Second, how to ensure the timely and accurate postoperative follow-up is very important and difficult in this study. We have established guidelines and training for research staff. Patient self reporting is another supplement.

In conclusion, the results of this methodologically rigorous trial are expected to provide not only clinical evidence of the effectiveness and safety of TEAS on GI function in patients after abdominal surgery but also demonstrate the mechanism of TEAS treatment on GI function.

## Trial status

The first participant was enrolled on September 1, 2019, and the first version was developed on August 20, 2019. The recruitment will be completed on December 31, 2020. Shown above is the third version whose protocol was revised for the following reasons: unspecific statistical methods; imprecise sample size calculation; and inappropriate descriptions. To date, 106 participants have been recruited. This trial is still ongoing.

## Supplementary information

**Additional file 1.** Standard Protocol Items: Recommendations for Interventional Trials (SPIRIT) 2013 Checklist.

## Data Availability

After the study is completed, the data will be open to the public through the Research Manager (ResMan) platform (http://www.medresman.org/login.aspx) within 6 months.

## Abbreviations

AE: Adverse event
CCK: Cholecystokinin
CNS: Central nervous system
DMC: Data Monitoring Committee
ERAS: Enhanced recovery after surgery
GI: Gastrointestinal
IBS: Irritable bowel syndrome
PGD: Postoperative gastrointestinal dysfunction
SP: Substance P
TEAS: Transcutaneous electrical acupuncture stimulation
VIP: Vasoactive intestinal polypeptide
